# Prognostic Association between Common Laboratory Tests and Overall Survival in Elderly Men with De Novo Metastatic Castration Sensitive Prostate Cancer: A Population-Based Study in Canada

**DOI:** 10.3390/cancers13112844

**Published:** 2021-06-07

**Authors:** Christopher J. D. Wallis, Bobby Shayegan, Scott C. Morgan, Robert J. Hamilton, Ilias Cagiannos, Naveen S. Basappa, Cristiano Ferrario, Geoffrey T. Gotto, Ricardo Fernandes, Soumyajit Roy, Krista L. Noonan, Tamim Niazi, Sebastien J. Hotte, Fred Saad, Huong Hew, Katherine F. Y. Chan, Laura Park-Wyllie, Shawn Malone

**Affiliations:** 1 Vanderbilt University Medical Center, Department of Urologic Surgery, Nashville, TN 37232-2765, USA; 2 St. Joseph’s Healthcare, McMaster University, Hamilton, ON L8N 4A6, Canada; shayeb@mcmaster.ca; 3 The Ottawa Hospital, University of Ottawa, Ottawa, ON K1H 8L6, Canada; smorgan@toh.ca (S.C.M.); icagiannos@toh.ca (I.C.); SMalone@toh.ca (S.M.); 4 Princess Margaret Cancer Centre, University of Toronto, Toronto, ON M5G 2M9, Canada; rob.hamilton@uhn.ca; 5 Cross Cancer Institute, University of Alberta, Edmonton, AB T6G 1Z2, Canada; naveen.basappa@albertahealthservices.ca; 6 Segal Cancer Centre, Jewish General Hospital, McGill University, Quebec, QC H3T 1E2, Canada; cristiano.ferrario@mcgill.ca; 7 Southern Alberta Institute of Urology, University of Calgary, Calgary, AB T2V 1P9, Canada; drgotto@gmail.com; 8 London Regional Cancer Program, London, ON N6A 5W9, Canada; ricardo.fernandes@lhsc.on.ca; 9 Radiation Oncology, Rush University Cancer Center, Chicago, IL 60612, USA; soumyajitroy8@gmail.com; 10 BC Cancer Agency, University of British Columbia, Surrey, BC V5Z 4E6, Canada; knoonan2@bccancer.bc.ca; 11 Jewish General Hospital, McGill University, Quebec, QC H3T 1E2, Canada; tniazi@jgh.mcgill.ca; 12 Juravinski Cancer Centre, McMaster University, Hamilton, ON L8V 5C2, Canada; hotte@hhsc.ca; 13 Centre Hospitalier de l’Université de Montréal, University of Montreal, Montréal, QC H2X 3E4, Canada; fredsaad@videotron.ca; 14 Janssen Inc., Toronto, ON M3C 1L9, Canada; hhew@its.jnj.com (H.H.); kchan@its.jnj.com (K.F.Y.C.); lparkwyl@ITS.JNJ.com (L.P.-W.)

**Keywords:** mCSPC, prognosticators, lab tests, OS, population-based

## Abstract

**Simple Summary:**

Despite significant advancements in the treatment of metastatic prostate cancer, a validated prognostic tool for patients with de novo metastatic castration-sensitive prostate cancer (mCSPC) is still lacking. Using population-based data from Ontario, Canada, we examined the prognostic association between common laboratory tests and survival for patients with mCSPC. These low-cost commonly available laboratory tests, including neutrophil-to-lymphocyte ratio, platelet-to-lymphocyte ratio, albumin, hemoglobin, PSA decrease and PSA nadir, were significantly associated with OS. They can provide important prognostic information and should be utilized more frequently among patients with newly diagnosed mCSPC.

**Abstract:**

De novo cases of metastatic prostate cancer (mCSPC) are associated with poorer prognosis. To assist in clinical decision-making, we aimed to determine the prognostic utility of commonly available laboratory-based markers with overall survival (OS). In a retrospective population-based study, a cohort of 3556 men aged ≥66 years diagnosed with de novo mCSPC between 2014 and 2019 was identified in Ontario (Canada) administrative database. OS was assessed by using the Kaplan–Meier method. Multivariate Cox regression analysis was performed to evaluate the association between laboratory markers and OS adjusting for patient and disease characteristics. Laboratory markers that were assessed include neutrophil-to-lymphocyte ratio (NLR), platelet-to-lymphocyte ratio (PLR), albumin, hemoglobin, serum testosterone and PSA kinetics. Among the 3556 older men with de novo mCSPC, their median age was 77 years (IQR: 71–83). The median survival was 18 months (IQR: 10–31). In multivariate analysis, a statistically significant association with OS was observed with all the markers (NLR, PLR, albumin, hemoglobin, PSA decrease, reaching PSA nadir and a 50% PSA decline), except for testosterone levels. Our findings support the use of markers of systemic inflammation (NLR, PLR and albumin), hemoglobin and PSA metrics as prognostic indicators for OS in de novo mCSPC.

## 1. Introduction

Prostate cancer (PCa) is the most common solid organ tumor in Canadian men [1,2,3]. Nearly a quarter of all PCa patients will have metastatic disease at some point in their disease trajectory, with a third of having metastatic disease at diagnosis (de novo) [1,4] which are associated with worse prognosis [5,6]. In the hormone-naïve setting, the median overall survival (OS) among patients with metastatic prostate cancer receiving conventional treatment (i.e., androgen-deprivation therapy (ADT)) is 36–40 months [4,5,7] and is reportedly much worse (<20 months) in the elderly population [8]. Recently, numerous studies have demonstrated benefits of ADT intensification regimens in mCSPC patients. The addition of chemotherapy (docetaxel) [7,9,10] or androgen receptor-axis-targeted therapies (abiraterone acetate + prednisone [11,12,13], apalutamide [14] and enzalutamide [15]) to ADT have been found to result in improved overall survival (OS) and progression-free survival (PFS).

As the treatment for mCSPC becomes more complex with emerging systemic and local therapy, common indicators of disease burden can assist in clinical decision-making and improve patient management. At present, management is tailored based on metastatic burden, as described in CHAARTED, and risk stratification, as based on LATITUDE criteria. A number of prognostic models have been developed for patients with metastatic PCa [16,17,18,19,20]. These include the Glass model (2003) [18], Gravis model (2015) [19] and, most recently, Akamatsu [20] model. However, these models have their own limitations. For example, the Glass model was built from a trial that was conducted in an era when use of PSA was uncommon. The Gravis model was based on a very small number of patients with relatively low discriminatory ability and the Akamatsu model was based on a retrospective review of a small number of patients with only Asian ethnicity. Consequently, these models should be validated in larger, more diverse, real-world populations [21]. Additionally, the prognostic values of other easily accessible laboratory parameters (e.g., markers of inflammation, testosterone levels, etc.) were unexplored in these models in de novo mCSPC, despite evidence supporting their prognostic ability in the metastatic castrate resistant prostate cancer (mCRPC) population [22,23,24].

We, therefore, performed a large population-based study on elderly patients aged 66 years and above with de novo mCSPC from a Canadian province to determine the prognostic association of several commonly available laboratory-based markers with overall survival (OS).

## 2. Materials and Methods

### 2.1. Study Design

A retrospective population-based cohort study was conducted by using province-wide linked administrative data.

### 2.2. Setting

Canada has a universal, publicly funded healthcare system where about 70% of total health expenditure comes from general taxation of the federal and 13 provincial/territorial governments [25,26]. Ontario is the most populous province in Canada, with almost 14.7 million residents, representing about 40% of the Canadian population [27]. In Ontario, cancer-care services are coordinated by Cancer Care Ontario, a government agency responsible for collecting cancer data, developing clinical standards, and planning and implementing cancer services through 14 regional cancer centers [28].

### 2.3. Data Sources

The patient-level dataset was created by using health administrative databases housed at IC/ES (www.ices.on.ca, accessed on 31 March 2020), an independent, non-profit research corporation funded by the Ministry of Health and Long-Term Care. The databases contain publicly funded administrative health service records for the Ontario population eligible for health coverage. They were linked by using encrypted patient-specific identifiers. Table A1 presents a description of databases used to create the study dataset.

### 2.4. Study Population

A cohort of de novo mCSPC patients in the province was defined among men aged ≥66 years who had metastatic PCa at the time of diagnosis (i.e., index date) between 1 January 2014 and 31 March 2020. Subjects were excluded if they were age ≤65, had a missing or invalid identification number, were not eligible for provincial health insurance coverage in the 2 years prior to diagnosis or had missing data for key exposure variables for analyses. Because the Ontario Drug Benefit (ODB) coverage starts at 65 years of age, we included patients aged ≥66 years to capture at least one year of full prescription data prior to the index date. This allowed for identification of an inception cohort and complete treatment ascertainment.

### 2.5. Outcome and Covariates

The primary end point used in the study was OS, defined as the time from PCa diagnosis to date of death from any cause. Patients without events were censored at their last date of follow-up. We evaluated the association of several exposure variables of interest with OS: markers of systemic inflammation (baseline neutrophil to lymphocyte ratio (NLR), platelet to lymphocyte ratio (PLR) and albumin), baseline hemoglobin, baseline testosterone levels and prostate-specific antigen (PSA) metrics (50% PSA decrease, PSA percentage change from baseline, PSA nadir <0.1 ng/mL). These exposure variables were selected because they are readily available from laboratory tests and have been implicated in the prognosis of prostate cancer [22,23,24,29,30]. Of note, all laboratory tests were performed in the 3 months preceding the index date. If the test was not available prior to the index date, then we used a test value available within 2 months following the index date but no more than 5 days after ADT initiation. In either case, the test value that was closest to the index date was selected. In a sensitivity analysis, we considered laboratory tests performed in the 6 months preceding the index date. As an exploratory analysis, we evaluated the association of these laboratory markers with time to emergence of castrate resistance.

The study population was described at baseline, using a set of variables that included sociodemographic characteristics (age, socioeconomic status, Local Health Integration Network (LHIN), rurality, PCa attributes such as PSA at diagnosis, Gleason score and health status (Charlson Comorbidity Index, history of key comorbid conditions other than PCa, including diabetes, myocardial infarction, congestive heart failure, and liver or kidney disease), as well as healthcare use (number of general practitioner (GP) visits, status of being a long-term care (LTC) resident and history of hospitalizations). Details on each variable are briefly presented in Table A2.

### 2.6. Statistical Analysis

Descriptive statistics (counts (%); mean and standard deviation (SD) or median with inter-quartile range (IQR)) were reported for the baseline patient characteristics. Median was used to describe years, as well as PSA, at diagnosis (due to its non-normal nature). Overall survival (OS) (median with IQR) for the entire cohort was calculated by using the Kaplan–Meier method.

Utilizing a complete case analysis, we fitted a Cox proportional hazard model to assess the association between each of the exposure variables and OS. Hazard ratios (HRs) and their associated 95% confidence intervals (CI) were computed. We first examined the relationship between each exposure lab test and overall survival by using restricted cubic splines (RCS) with 5 knots to assess for non-linearity. We identified significant non-linearity, and, to provide clinically interpretable estimates, the exposure variables were categorized into quintiles, with the lowest quintile (Q1) used as the reference. We also tested a binary categorization of the non-ratio type of exposure variables, i.e., “normal vs. below normal” for albumin, hemoglobin, testosterone and “yes vs. no” for 50% PSA decrease and PSA nadir <0.1 ng/mL. The models were compared by using negative log-likelihood (NLL) values. Results for all the models were reported. The assumption of proportionality was tested by the examination of Schoenfeld residuals.

Each Cox model was adjusted for baseline characteristics. All the characteristics were introduced to the models as dichotomized or categorical independent variables, with the exception of GP visits (continuous) and PSA (splines). For the latter, the baseline PSA values were transformed by using restricted cubic splines (RCS) with 5 knots to account for non-linearity. Table A3 provides more detail on categorization of the independent covariates, including the exposure variables. There was no evidence of multicollinearity between the covariates in the model. All analyses were conducted in SAS. The study received research ethics approval from Advarra IRB (Pro00037601).

## 3. Results

### 3.1. Patient Characteristics

From 2014 to 2019, we identified 3556 patients of age 66 years or older who presented with de novo metastatic PCa. The baseline characteristics are summarized in Table 1. The median age of the patients was 77 years (IQR: 71–83). Table 2 provides a distribution of patients’ laboratory tests (i.e., exposure variables) across the quintiles. The total number of patients with corresponding lab results available for modeling ranged from 290 (testosterone) to 2775 (PSA).

### 3.2. Association between Laboratory Tests and Overall Survival 

The median survival of the entire population of de novo mCSPC patients was 18 months (IQR: 10–31). The adjusted Cox regression analyses showed that all exposure variables, except serum testosterone, were significantly associated with OS (Table 3 and Table 4).

#### 3.2.1. Neutrophil to Lymphocyte (NLR) and Platelet to Lymphocyte Ratios (PLR)

After adjusting for the baseline characteristics, men with higher NLR (quintile 5) had significantly greater relative risk of mortality than those with lower NLR (quintile 1) (hazard ratio (HR) _Q5 vs. Q1_: 1.55; 95% confidence intervals (CI): 1.27–1.90). Similarly, patients with high PLR (quintile 5) had increased risk of mortality compared to those with low PLR (quintile 1) (HR_Q5 vs. Q1_: 1.36; 95% CI: 1.11–1.65).

#### 3.2.2. Albumin

After adjusting for confounders, patients with high albumin levels (quintile 5), had significantly decreased risk of mortality (HR_Q5 vs. Q1_: 0.48; 95% CI: 0.36–0.63) compared to men with low albumin (quintile 1).

#### 3.2.3. Hemoglobin 

Similarly, hemoglobin levels were associated with OS. Men with high hemoglobin levels (quintile 5) had a significantly decreased risk of mortality compared to patients with low hemoglobin (quintile 1) (HR_Q5 vs. Q1_: 0.42; 95% CI: 0.33–0.52).

#### 3.2.4. PSA, % Drop from Baseline

Compared with patients with the steeper decline in PSA from the baseline within 3 months (quintile 5), patients with a flatter PSA decline (quintile 1) had a significantly greater risk of dying (HR_Q5 vs. Q1_: 0.21; 95% CI: 0.18–0.26). Patients who were able to reach the prespecified lowest PSA (<0.1 ng/mL) and patients who had at least a 50% decline in PSA at 3 months had a significantly decreased risk of overall mortality: HR_PSA nadir_: 0.27 (95% CI: 0.22–0.32) and HR_50%PSA_: 0.26 (95% CI: 0.22–0.31), respectively.

#### 3.2.5. Testosterone

There was no observed association between baseline testosterone levels and OS (HR_Q5 vs. Q1_: 0.99; 95% CI: 0.54–1.82).

Compared to the model based on binary categorization of the variables (based on lower limit of normal), the quintile-based stratification showed superior results for albumin (negative log-likelihood (NLL): 7640.6 vs. 7624.8, *p* = 0.012) and hemoglobin (NLL: 14310.0 vs. 14291.8, *p* < 0.001) but no difference was seen for testosterone (*p* = 0.327) (Table A4). For exploratory analysis, no significant associations were observed between the exposure variables and the time to castrate resistance. 

## 4. Discussion

In this study, we examined the prognostic value of several commonly used laboratory markers in a large cohort of patients aged 66 years and older with de novo mCSPC. The results indicate that patients with higher NLR or PLR had a 55% and 36% relative increase in the risk of mortality compared to those with low NLR or PLR, respectively. In contrast, patients with higher levels of albumin and patients with higher levels of hemoglobin had a 52% and 58% reduced risk of death respectively, compared to those with lower values. Patients with a faster rate of decline in PSA levels and those who ultimately achieved the PSA nadir were found to have superior OS compared to those with a slower decline in PSA levels and those who failed to achieve the predefined PSA nadir. We did not find any association of testosterone levels with OS; however, this comparison was limited by a relatively smaller sample size and inter-assay variability. Moreover, the observed lack of association between the markers and time to castration resistance suggests that other PCa disease-specific characteristics may need to be explored for prognostic use of disease progression among this population.

Emerging evidence has demonstrated the pivotal role of chronic inflammation on tumorigenesis and disease progression. Lymphocytopenia and higher platelet counts trigger cancer progression and hematogenous dissemination. NLR and PLR, as biomarkers for systemic inflammatory response, have been found to be associated with poor prognosis in mCRPC and mCSPC [31,32,33]. Our findings concur with these results. Further, our finding of poor overall survival in patients with hypoalbuminemia could be attributed to a number of factors. Hypoalbuminemia is essentially caused by chronic systemic inflammatory activity including ageing itself. Hypoalbuminemia results in diminished muscle mass; it bears detrimental impact on cognitive and immune function and, consequently, leads to diminished life expectancy [34]. In contrast to our findings, Akamatsu et al. did not find any association of albumin levels with OS. The differences in study populations including age, different jurisdictions and different healthcare systems could be some of the plausible explanations behind these conflicting observations.

Anemia in men with advanced prostate cancer may be caused by several factors, including androgen deprivation, decline in nutritional status, bone marrow infiltration, treatment-related toxicity and the chronic inflammatory state. Low levels of hemoglobin have been reported to portend worse overall and cancer-specific survival in patients with mCSPC [35,36] independent of the metastatic burden. Our findings are in agreement with these results. This association could be explained by anemia-induced dimerization of hypoxia-inducible factor 1 alfa (HIF-1α). HIF-1α triggers expression of various oncogenes, conferring resistance to chemotherapy and radiotherapy and inducing cancer progression [37,38,39].

The association of PSA kinetics with OS is controversial. Although the Glass model showed PSA to be an important prognostic factor, Akamatsu et al. did not include PSA in their final model. Petrylak et al. showed that PSA-level declines of at least 20%–40%, in addition to a PSA velocity at 3 months after beginning treatment with docetaxel, were associated with overall survival [40]. A recent post hoc analysis of LATITUDE study showed significant correlation of time-to-PSA progression with radiographic progression-free survival and OS [41]. Patients reaching PSA ≤0.1 ng/mL within 6 months had a significantly longer radiographic progression-free survival or OS. Additionally, more than 50% response in PSA was associated with decreased risk of death (HR, 0.44; 95% CI 0.27–0.70). These findings are concordant with our results. Overall, these findings suggest that early PSA kinetics after treatment could reflect a long-term overall outcome in this patient population.

Our study is subject to a number of limitations. First, the retrospective nature from which these prognostic associations were derived should be validated with results from prospective de novo mCSPC studies. Second, the lack of radiographic data to differentiate between patients with high vs. low volume of metastatic burden and the lack of information on these laboratory variables in a substantial proportion of patients were limiting factors. The overall frequency of PSA measurements in an individual patient was low, leading to missed opportunity to acquire valuable information. The frequency and timing of PSA determination also varied among treating oncologists. Our findings should be considered in light of these confounders. Third, our study cohort was relatively older compared to those included in the randomized controlled trials. Moreover, this analysis is based on patients with heterogeneous clinical backgrounds not controlled for in the context of a prospective clinical trial. Finally, factors such as patient or clinician preference, baseline psychological distress, anxiety and health-related quality of life, which might affect treatment choice and overall survival, were not captured in our study [42].

## 5. Conclusions

Our study confirmed the potential utility of markers of systemic inflammation such as NLR, PLR and albumin to serve as prognostic indicators for overall survival in a large cohort of de novo mCSPC. Our findings also supported the use of PSA metrics as prognostic indicators for survival in the de novo mCSPC. Overall, these findings suggest that these biomarkers should be considered among other clinical features to tailor treatment decision in men with de novo metastatic castrate sensitive prostate cancer.

## Figures and Tables

**Table 1:** Baseline characteristics.

Parameter	Value
**Patient demographics**	
**Age categories**	
Median (IQR), years	77 (71-83)
Age 66–75, *n* (%)	1576 (44.3%)
Age 76–85, *n* (%)	1382 (38.9%)
Age > 85, *n* (%)	598 (16.8%)
**Rurality**	
Non-rural, *n* (%)	3156 (88.8%)
**Medical care and comorbidity**	
Comorbidity (Charlson Index), Mean ± SD	0.33 ± 0.99
Number of GP visits, Mean ± SD	9.23 ± 8.49
Hospitalization, *n* (%)	593 (16.7%)
Ever LTC resident, *n* (%)	50 (1.4%)
Diabetes, *n* (%)	261 (7.3%)
History of MI, *n* (%)	87 (2.4%)
History of CVA, *n* (%)	67 (1.9%)
History of CHF, *n* (%)	252 (7.1%)
History of COPD, *n* (%)	211 (5.9%)
History of HTN, *n* (%)	362 (10.2%)
History of arrhythmia, *n* (%)	52 (1.5%)
History of dementia, *n* (%)	319 (9.0%)
History of liver disease, *n* (%)	37 (1.0%)
History of renal disease, *n* (%)	311 (8.7%)
**Prostate cancer characteristics**	
PSA at diagnosis (3 months)	-
PSA test, *n* (%)	2775 (78.0%)
Median (IQR)	85 (24–346)
**Biopsy Gleason Score**	
<7, *n* (%)	13 (0.4%)
7, *n* (%)	301 (8.5%)
>7, *n* (%)	1426 (40.1%)
Unknown, *n* (%)	1816 (51.1%)
**Systemic therapies received**	
Conventional ADT, *n* (%)	2794 (78.6%)
ADT plus Docetaxel, *n* (%)	399 (11.2%)
ADT plus AA + P, *n* (%)	52 (1.5%)
Non-ADT, *n* (%)	311 (8.7%)

PSA, prostate-specific antigen; HTN, hypertension; IQR, inter-quartile range; SD, standard deviation; COPD, chronic obstructive pulmonary disorder; CHF, congestive heart failure; CVA, cerebrovascular accidents; MI, myocardial infarction; LTC, long-term care; ADT, androgen deprivation therapy; AA + P, abiraterone acetate plus prednisone.

**Table 2:** Quintile values of laboratory tests (exposure variables).

Laboratory Test	Parameter	Quintile 1	Quintile 2	Quintile 3	Quintile 4	Quintile 5	Total
Neutrophil to lymphocyte ratio	No. of patients	*n* = 410	*n* = 411	*n* = 407	*n* = 411	*n* = 410	*n* = 2049
	Mean ± SD	1.4 ± 0.4	2.3 ± 0.2	3.1 ± 0.2	4.2 ± 0.5	8.9 ± 6.1	4.0 ± 3.8
Platelet to lymphocyte ratio	No. of patients	*n* = 406	*n* = 406	*n* = 406	*n* = 407	*n* = 406	*n* = 2031
	Mean ± SD	69.2 ± 22.2	112.1 ± 9.5	145.1 ± 10.0	190.2 ± 17.5	324.3 ± 123.4	168.2 ± 104.4
Albumin (g/L)	No. of patients	*n* = 222	*n* = 205	*n* = 221	*n* = 178	*n* = 241	*n* = 1067
	Mean ± SD	28.9 ± 3.7	35.5 ± 1.1	39.0 ± 0.8	41.5 ± 0.5	44.7 ± 1.7	37.9 ± 5.9
Hemoglobin (g/L)	No. of patients	*n* = 404	*n* = 414	*n* = 404	*n* = 425	*n* = 418	*n* = 2,065
	Mean ± SD	95.6 ± 11.7	117.9 ± 4.4	130.4 ± 2.8	140.6 ± 2.8	154.6 ± 7.6	128.1 ± 21.2
Testosterone (nmol/L)	No. of patients	*n* = 59	*n* = 56	*n* = 59	*n* = 58	*n* = 58	*n* = 290
	Mean ± SD	0.3 ± 0.2	3.4 ± 2.0	8.7 ± 1.0	12.8 ± 1.4	21.4 ± 6.1	9.3 ± 8.0
PSA (nmol/L)	No. of patients	*n* = 555	*n* = 555	*n* = 556	*n* = 554	*n* = 555	*n* = 2,775
	Mean ± SD	9.7 ± 4.7	32.1 ± 9.8	89.2 ± 25.5	270.8 ± 95.2	1748.1 ± 2116.3	429.9 ± 1157.3

**Table 3:** Cox proportional hazards model of time to death by laboratory tests stratified by quintiles (Q).

Characteristics	Adjusted HR (95% CI) ^@^			
	Q2 vs. Q1 *	Q3 vs. Q1 *	Q4 vs. Q1 *	Q5 vs. Q1 *
NLR	1.08 (0.88–1.31)	1.08 (0.88–1.31)	1.44 (1.18–1.75)	1.55 (1.27–1.90)
PLR	0.95 (0.77–1.16)	1.08 (0.88–1.32)	1.23 (1.01–1.50)	1.36 (1.11–1.65)
Albumin	0.77 (0.60–0.99)	0.65 (0.51–0.84)	0.55 (0.42–0.73)	0.48 (0.36–0.63)
Hemoglobin	0.71 (0.59–0.85)	0.56 (0.47–0.69)	0.42 (0.34–0.52)	0.42 (0.33–0.52)
Testosterone	1.56 (0.89–2.74)	0.78 (0.43–1.42)	1.12 (0.61–2.08)	0.99 (0.54–1.82)
PSA% drop from baseline within 3 months	0.50 (0.43–0.59)	0.38 (0.32–0.46)	0.26 (0.21–0.32)	0.21 (0.18–0.26)

* Q1 is the reference quintile; Q5 represents the highest value. ^@^ The models were adjusted for baseline variables: age, socioeconomic status, LHIN, rurality, PSA at diagnosis, Gleason score, Charlson Comorbidity Index, history of diabetes, myocardial infarction, congestive heart failure, cardio-vascular accidents, liver or kidney disease, hypertension, chronic obstructive pulmonary disease, arrhythmia and dementia; number of GP visits, status of a LTC resident and history of hospitalizations.

**Table 4:** Cox proportional hazards model of time to death by laboratory markers stratified into binary variables by lower limit of normal level (binary categorization).

Characteristics	Value	Adjusted HR (95% CI) ^@^	*p*-Value
Albumin	Normal (34–54 g/L) vs. Below (<34 g/L)	0.60 (0.49–0.74)	<0.001
Hemoglobin	Normal (130–175 g/L) vs. Below (<130 g/L)	0.55 (0.48–0.63)	<0.001
Testosterone	Normal (9.5–30 nmol/L) vs. Below (<9.5 nmol/L)	0.78 (0.52–1.17)	0.2288
Achieve PSA nadir (<0.1 ng/mL)	Yes vs. No	0.27 (0.22–0.32)	<0.001
50% PSA decline at 3 months	Yes vs. No	0.26 (0.22–0.31)	<0.001

^@^ The models were adjusted for baseline variables: age, socioeconomic status, LHIN, rurality, PSA at diagnosis, Gleason score, Charlson Comorbidity Index, history of diabetes, myocardial infarction, congestive heart failure, cardio-vascular accidents, liver or kidney disease, hypertension, chronic obstructive pulmonary disease, arrhythmia and dementia; number of GP visits, status of a LTC resident and history of hospitalizations.

## Data Availability

Health administrative databases for Ontario, Canada, are housed at IC/ES (www.ices.on.ca, accessed on 31 March 2020).

## Appendix

**Table A1:** Description of databases that informed the development of the study dataset.

**Ontario Cancer Registry (OCR)**
The OCR is collected by Cancer Care Ontario and contains information on all Ontario residents who have been newly diagnosed with cancer (“incidence”) or who have died of cancer (“mortality”). All new cases of cancer are registered, except non-melanoma skin cancer.
**Registered Person Database (RPDB)**
The RPDB provides basic demographic information (age, sex, location of residence, date of birth and date of death for deceased individuals) for those issued an Ontario health insurance number. The RPDB also indicates the time periods for which an individual was eligible to receive publicly funded health insurance benefits and the best known postal code for each registrant on July 1st of each year.
**Continuing Care Reporting System (for Chronic Care) (CCRS)**
The CCRS database is compiled by the Canadian Institute for Health Information and contains demographic, clinical, functional, and resource utilization information for individuals receiving facility-based continuing care (also known as extended, auxiliary, or complex chronic care) in Ontario hospitals and residential care providing 24 h nursing services (i.e., nursing home). Clinical assessment data (on the physical, functional, cognitive and social domains of health) are ascertained by using the Resident Assessment Instrument-Minimum Data Set (RAI-MDS) version 2.0, which is administered by trained healthcare professionals.
**Ontario Congestive Heart Failure dataset (CHF)**
The Ontario Congestive Heart Failure Database is an ICES-derived cohort that was created by using a definition of ≥2 physician billing claims with a diagnosis of CHF (OHIP diagnosis code 428) and/or ≥1 inpatient hospitalization or same-day surgery record with a diagnosis of CHF (ICD-9 diagnosis code 428; ICD-10 diagnosis code I50; in the primary diagnostic code space) in a two-year period applied to hospitalization (DAD), same-day surgery (SDS), and physician billing claims (OHIP) data to determine the diagnosis date for incident cases of CHF in Ontario.
**Ontario Chronic Obstructive Pulmonary Disease Dataset (COPD)**
The Ontario COPD Database is an ICES-derived cohort that is created by using two separate algorithms applied to inpatient hospitalization (DAD), same-day surgery (SDS) records, and physician billing claims (OHIP) data to determine the diagnosis date for incident cases of COPD in Ontario. In an algorithm which maximizes sensitivity, the definition for COPD is any physician billing claim with a diagnosis for COPD (OHIP diagnosis codes 491, 492 and 496) or any inpatient hospitalization or same-day surgery record with a diagnosis for COPD (ICD-9 diagnosis codes 491, 492, and 496; ICD-10 diagnosis codes J41–J44; in any diagnostic code space).
**Discharge Abstract Database (DAD)**
The DAD is compiled by the Canadian Institute for Health Information and contains administrative, clinical (diagnoses and procedures/interventions), demographic, and administrative information for all admissions to acute care hospitals, rehab, chronic, and day surgery institutions in Ontario. At ICES, consecutive DAD records are linked together to form “episodes of care” among the hospitals to which patients have been transferred after their initial admission.
**Ontario Hypertension Dataset (HYPER)**
The Ontario Hypertension Database is an ICES-derived cohort and created by using a definition of ≥2 physician billing claims with a diagnosis of hypertension (OHIP diagnosis codes: 401–405) and/or ≥1 inpatient hospitalization or same-day surgery record with a diagnosis of hypertension (ICD-9 diagnosis codes: 401–405; ICD-10 diagnosis codes: I10-I13, I15; in any diagnostic code space) in a two-year period applied to hospitalization (DAD), same-day surgery (SDS), and physician billing claims (OHIP) data to determine the diagnosis date for incident cases of hypertension in Ontario. Physician claims and hospitalizations with a diagnosis of hypertension occurring within 120 prior to and 180 days after a gestational hospitalization record are excluded.
**National Ambulatory Care Reporting System (NACRS)**
The NACRS is compiled by the Canadian Institute for Health Information and contains administrative, clinical (diagnoses and procedures), demographic, and administrative information for all patient visits made to hospital- and community-based ambulatory care centers (emergency departments, day surgery units, hemodialysis units, and cancer care clinics). At ICES, NACRS records are linked with other data sources (DAD and OMHRS) to identify transitions to other care settings, such as inpatient acute care or psychiatric care.
**Ontario Diabetes Dataset (ODD)**
The Ontario Diabetes Database is an ICES-derived cohort and is created by using algorithms applied to inpatient hospitalization (DAD) records, same-day surgery (SDS) records and physician billing claims (OHIP) data to determine the diagnosis date for incident cases of diabetes in Ontario. For adults aged 19 years and greater, the definition for diabetes is two physician billing claims with a diagnosis for diabetes (OHIP diagnosis code 250) or one inpatient hospitalization or same-day surgery record with a diagnosis for diabetes (ICD-9 diagnosis code 250; ICD-10 diagnosis codes E10, E11, E13 and E14; in any diagnostic code space) within a 2-year period. Physician claims and hospitalizations with a diagnosis of diabetes occurring within 120 prior to and 180 days after a gestational hospitalization record were excluded.
**Ontario Health Insurance Plan Claims Database (OHIP)**
OHIP claims data received by ICES contain most claims paid for by the Ontario Health Insurance Plan. The data cover all healthcare providers who can claim under OHIP (this includes physicians, groups, laboratories and out-of-province providers). Approximately 95% of specialists and 50% of primary care physicians receive the majority of their income from fee-for-service (FFS). However, all physicians, with the exception of the few hundred family physicians who work in Community Health Centres, are required to submit shadow billings for their non-FFS services. A shadow-billing claim is identical to an FFS claim except that the payment amount is $0.00. Shadow-billings have an explain code of “I2”. Physicians are often provided with cash incentives to encourage them to shadow-bill. Requiring physicians to shadow-bill helps to ensure that the OHIP data accurately (more-or-less) reflect the utilization of physician services in Ontario.
**Ontario Myocardial Infarction Dataset (OMID)**
The Ontario Myocardial Infarction Database is an ICES-derived cohort and contains records of all inpatient hospital admissions for acute MIs (ICD-9 diagnosis code 410; ICD-10 diagnosis code I21; in the primary diagnostic code space) in Ontario since 1991. These admissions are ascertained by using the DAD and exclude in-hospital events and admissions where there had been a previous discharge for acute myocardial infarction in the previous year. This cohort of patients with acute MI hospital admissions is linked with hospitalization (DAD), same-day surgery (SDS) and physician-billing-claims data (OHIP) to create indicators of hospital readmission after discharge and receipt of cardiac procedures during and after the initial hospital admission.
**Ontario Laboratories Information System (OLIS)**
Starting in 2012 all Public Health Ontario laboratories joined OLIS. As of August 2016, OLIS has completed connections with additional hospital laboratories in 13 out of 14 LHINs. As of February 2017, Hamilton Health Sciences is now fully contributing lab results into OLIS. The OLIS library at ICES consists of three distinct datasets: (1) lab orders contain the order-level information, including patient demographics and provider information; (2) test requests contain the test ID code (called Test Request Code) and specimen information, in addition to ordering, performing and reporting facilities among other variables; and (3) observations contain the test result information, including the result ID code (called Observation Code), values and units.
**New Drug Funding Program (NDFP)**
The New Drug Funding Program (NDFP) is one of four publicly funded drug programs under the Ontario Public Drug Programs (OPDP). Administered by Cancer Care Ontario, the NDFP funds new, and often very expensive, cancer drugs. The program was created in 1995 to ensure that Ontario patients have equal access to high-quality intravenous (IV) cancer drugs.
**Cancer Activity Level Reporting (ALR)**
Activity Level Reporting (ALR) data represent the basic set of data elements required to produce the quality, cost and performance indicators for the cancer system. The data elements constitute patient-level activity within the cancer system focused on radiation and systemic therapy services and outpatient oncology clinic visits. These data are also a key component of the Ontario Cancer Registry (OCR), which registers every malignant neoplasm diagnosed in Ontario.

**Table A2:** Definition of variables to describe baseline characteristics.

**Category**	** Description**
**Demographics**	- Age at index date (from RPDB) - Socioeconomic status at index date (neighborhood income quintile) - eographic region at index date (LHIN, rural vs. non-rural)
**Prostate cancer characteristics**	- PSA (median, IQR) immediately prior to prostate cancer diagnosis (must be within 6 months prior to diagnosis; if none available prior to diagnosis, check for one within 2 months following diagnosis; if none available by these criteria, mark as missing), using LOINC codes LOINC 19197-3, 2857-1, 35741-8) - Stage (OCR—best_stage_grp) - Grade (Gleason score)
**General medical care**	- Comorbidity—Charlson (CCI; 2-year look-back, using DAD and SDS data, including index) - Number of GP visits (spec = 00) in year prior to diagnosis - Hospitalization (inpatient for any reason) in year prior to diagnosis (yes/no) - Ever resident of long-term care in year prior to diagnosis (yes/no)
**Specific comorbidity**	- Diabetes diagnosis (ODD) (up to 2017) - Myocardial infarction in 5 years prior to diagnosis (OMID) Since OMID only up to date until 2016, use OMID and DADDefinition: either flagged in OMID and/or Main diagnosis hospitalization with ICD-10 I21 with no prior hospitalization for MI 1 year prior - Cerebrovascular accident in 5 years prior to diagnosis (NACRS, DAD) ICD-10: I63.x,167.81, I67.82 DAD—Most responsible diagnosis NACRS—Main diagnosis - Congestive heart failure (CHF ICES cohort) (up to 2017) - COPD (COPD ICES cohort) (up to 2017) - Hypertension (Hyper ICES cohort) (up to 2017) - HED/hospitalization for arrhythmia in year prior to diagnosis (NACRS, DAD) ICD-10: I44.x, I45.x, I47.x, I48.x, I49.x DAD—Most responsible diagnosis NACRS—Main diagnosis - Diagnosis of dementia within 5 years of diagnosis (NACRS, DAD, OHIP) ICD-10: F01.x, F02.x, F03.x, G30.X, G31.83, DXCODE: 290 DAD—Most responsible diagnosis NACRS—Main diagnosis OHIP—DXCODE - Diagnosis of liver disease within 5 years prior to diagnosis (NACRS, DAD, OHIP) ICD-10: K70.x, K71.x, K72.x, K73.x, K74.x, K75.2, K75.3, K75.4, K75.8, K75.9, K76.9 DXCODE: 571 DAD—Most responsible diagnosis NACRS—Main diagnosis OHIP—DXCODE - Diagnosis of renal disease within 5 years prior to diagnosis (NACRS, DAD, OHIP) ICD-10: N18.x, N19 DXCODE: 585 DAD—Most responsible diagnosis NACRS—Main diagnosis OHIP—DXCODE

**Table A3:** Categorization of the independent covariates.

**Quintile**	**No. of Patients**	**Min Value**	**Max Value**
Neutrophil to lymphocyte ratio value			
1	358	0	1.9
2	372	1.9	2.7
3	360	2.7	3.5
4	341	3.5	5.1
5	333	5.1	63.2
Platelet to lymphocyte ratio value			
1	349	3.6	95.7
2	358	95.8	129.2
3	361	129.2	163.3
4	352	163.5	224
5	333	224.4	1062.5
Albumin value			
1	185	17	33
2	177	33.2	37.5
3	189	38	40
4	166	40.1	42.7
5	220	43	53
Hemoglobin value			
1	316	46	109
2	354	110	125
3	355	126	135
4	376	136	145
5	376	146	183
Serum testosterone value			
1	59	0.1	0.6
2	55	0.7	6.6
3	57	6.8	10.3
4	58	10.4	15.2
5	57	15.3	46.8
PSA response (% drop from baseline) in absolute value			
1	475	0	85.7
2	475	85.7	96.8
3	475	96.8	99.2
4	475	99.2	99.9
5	475	99.9	62547.1

**Table A4:** Comparison of negative log-likelihood values of quintile-based model vs. model based on binary categorization.

**Characteristics**	**Albumin**	**Hemoglobin**	**Testosterone**
Negative LLV for model using categorical classification (quintiles)	7624.8	14,291.8	1466.1
Negative LLV for model using bivariate classification	7640.6	14,310.0	1469.6
Difference of LLV in 2 models	15.8	18.21	3.45
DF	3	2	3
*p*-value	0.0012	<0.001	0.327

The models were adjusted for baseline variables; LLV—log-likelihood value.
